# Redox status of biomarkers in serum of dogs with hypothyroidism and its treatment with levothyroxine sodium

**DOI:** 10.3389/fvets.2025.1490369

**Published:** 2025-04-04

**Authors:** Ling Zhu, Jinge Han, Halihaxi Bahetijiang, Gang Liu, John P. Kastelic, Xueying Zhou, Bo Han

**Affiliations:** ^1^College of Veterinary Medicine, China Agricultural University, Beijing, China; ^2^College of Animal Science and Veterinary Medicine, Tianjin Agricultural University, Tianjin, China; ^3^Faculty of Veterinary Medicine, University of Calgary, Calgary, AB, Canada

**Keywords:** canine hypothyroidism, antioxidant status, thyroxin, thyroid stimulating hormone, levothyroxine sodium therapy

## Abstract

Information regarding canine hypothyroidism in China remains limited, particularly regarding the redox status of affected dogs. Therefore, the objective of this study was to evaluate the redox status of dogs with hypothyroidism and observe how it changed after levothyroxine replacement therapy. A total of 10 healthy dogs (control group) and 10 dogs with hypothyroidism (treatment group) from China Agricultural University Veterinary Teaching Hospital were included in the study. The redox status was assessed in all 20 dogs. Thyroid profiles such as thyroxine (T4), free thyroxine (fT4), and thyroid-stimulating hormone (TSH) and biomarkers of oxidative stress, including superoxide dismutase (SOD), catalase (CAT), glutathione peroxidase (GSH-px), and malondialdehyde (MDA), were assessed on day 0 in all dogs and on days 14 and 45 post-levothyroxine sodium treatment in dogs with hypothyroidism. Dogs with hypothyroidism had decreased levels of serum T4 and fT4 and increased levels of serum TSH. Based on SOD, CAT, GSH-px, and MDA, dogs with hypothyroidism had oxidative stress. Following 45-day treatment with levothyroxine sodium (0.02 mg/kg orally twice daily), antioxidant parameters improved progressively: SOD increased from 60.2 to 76.7 U/mL (Day 0-14) and further to 83.3 U/mL (Day 45); CAT from 105.9 to 115.5 mU/mL (Day 0-14) reaching 132.9 mU/mL (Day 45); GSH-Px from 10.2 to 8.4 U/mL (Day 0-14) then 9.7 U/mL (Day 45), while MDA decreased from 8.2→6.8→4.4 μmol/L correspondingly. In conclusion, increased serum TSH and MDA concentrations, in addition to decreased serum T4 and fT4 concentrations and decreased SOD and CAT activities, indicated that there was oxidative stress in dogs with hypothyroidism. However, there were significant improvements in the redox status of biomarkers in the serum of dogs with hypothyroidism after treatment with levothyroxine sodium (0.02 mg/kg twice daily) for 45 days.

## 1 Introduction

The overall prevalence of canine hypothyroidism is 1.89% in the United States (1, 2), 2.7% in Norway (3), 3.9% in Germany (4), and 7.2% in Sweden (5). Hypothyroidism is characterized by a reduction in total thyroxine (T4) and triiodothyronine (T3) production by the thyroid gland. Clinical signs are often associated with a decreased basal metabolic rate and skin lesions (6). Thyroid hormone concentrations are closely associated with oxidative stress as they affect the number and activity of mitochondrial respiratory chain reactions, promote metabolic effects, and increase oxygen consumption, respiration rate, energy expenditure, and production of reactive oxygen species (ROS) (7). However, when the thyroid gland is in a pathological state, the oxidative and antioxidant balance is disrupted, with changes in the cellular redox environment causing oxidative stress (8). In humans, one study reported hypothyroidism induced a hypometabolic state, reducing ROS production and protecting the body from oxidative stress (9), whereas another study reported that hypothyroidism decreased antioxidant capacity and ROS scavengers, leading to ROS accumulation and oxidative stress (10). In humans, biomarkers of oxidative stress associated with hypothyroidism include decreased antioxidants and increased lipid peroxidation (8, 11, 12); however, these changes have been improved with the use of levothyroxine sodium (13). Regardless, there are few studies on canine hypothyroidism and oxidative stress. Therefore, the objective of this study was to evaluate the serum redox status of dogs with hypothyroidism and to observe any changes following levothyroxine replacement therapy.

## 2 Materials and methods

### 2.1 Statement of ethics

The study was reviewed and approved by the China Agricultural University Animal Care Committee and the Standard Biosafety and Security Committee (Protocol SYXK, 2022–0008).

### 2.2 Animals and procedures

This study was conducted from January 2023 to January 2024 at the Veterinary Teaching Hospital of China Agricultural University. A total of 30 dogs were included in the study for suspected hypothyroidism based on physical examinations. However, dogs with chronic kidney disease, Cushing’s disease, or other health problems were excluded from the study. Therefore, 10 dogs with a primary clinical diagnosis of hypothyroidism were designated as a treatment group, with inclusion criteria of adult dogs >1 year of age without any co-morbidities that could significantly influence hypothyroidism or survival and not received any medications in the last 6 months. A comprehensive physical examination was conducted on each dog, and a complete history was documented, including their diet over the past year. The body condition score (BCS) was assessed using a nine-point evaluation system (14), and based on the BCS, dogs were allocated into three groups: normal weight (BCS 4–5/9 points), overweight (BCS 6–7/9 points), and obese (BCS 8–9/9 points) (14). Complete blood count (CBD) and serum biochemistry tests were performed to detect comorbidities and to ensure suitability for treatment. In addition, a control group of 10 healthy dogs was identified during routine vaccinations with the owners’ consent, with the inclusion criteria of adult dogs >1 year old and no medication history in the last 6 months. The selected control dogs did not have any complications, such as chronic kidney disease, Cushing’s disease, or other health problems.

### 2.3 Blood sample collection

On days 0, 14, and 45, blood samples (1.5 mL) were collected in tubes containing EDTA for a complete blood count (CBC). Additionally, samples of 5 mL were collected in tubes without any additives and were kept at room temperature (22–25°C) for ≥ 30 min. The tubes without additives were centrifuged at 3,170 *g* for 5 min to separate the serum.

### 2.4 Methods

Breed, age, sex, weight, medical history, and medication history were recorded. A Sysmex XN-1000 V automated hematology analyzer (Sysmex Corporation, Kobe, Japan) was used to conduct a CBC, and a Hitachi 7,600–020 Automatic Bio-Chemical Analysis Instrument (Hitachi High-Technologies Corporation, Ibaraki, Japan) was used for serum biochemistry. The serum activities of superoxide dismutase (SOD), catalase (CAT), glutathione peroxidase (GSH-px), and malondialdehyde (MDA) concentration were measured (in triplicate) using their respective assay kits (Shanghai Biyuntian Biotechnology Co., Ltd., Shanghai, China), according to manufacturer’s instructions, and were assessed on a DNM-9602 microplate reader at wavelengths of 450 and 340 nm. The serum concentrations of thyroxine (T4), free thyroxine (fT4) and thyroid-stimulating hormone (TSH) were measured using a Cobas® e 411 analyzer (Roche, Germany) with kits from Roche Diagnostics International Ltd., Mannheim, Germany for T4 and fT4 and Vcheck cTSH, BIONOTE, Gyeonggi-do, Korea for TSH. Based on clinical signs, physical examinations, and laboratory tests, dogs were segregated into two groups: healthy dogs were assigned to a control group, while dogs diagnosed with hypothyroidism were placed in a treatment group. Dogs with hypothyroidism were given levothyroxine sodium at a dosage of 0.02 mg/kg orally, twice daily. The redox status of biomarkers was assessed in all dogs on day 0 and on days 14 and 45 after the initiation of levothyroxine sodium treatment in the treatment group.

### 2.5 Statistical analyses

The results were expressed as mean ± standard deviation (SD) derived from at least three independent experiments. GraphPad Prism 8.0 (GraphPad Software, Inc., San Diego, CA, USA) was used for data analysis, and statistical significance was assessed using SPSS 26.0 (IBM Corp., Armonk, NY, USA). Statistical differences were assessed using one-way analysis of variance (ANOVA). Spearman’s correlation analyses were performed, and correlation coefficients are represented as *r*-values, with −1 < *r* < 0 indicating a negative correlation and 0 < *r* < 1 indicating a positive correlation. Significance levels were indicated as follows: *p < 0.05 and **p < 0.01.

## 3 Results

### 3.1 Information and clinical signs

The age of the control group ranged from 4 to 13 years with a mean age of 7.6 years and a mean BCS of 5.9/9 (Table 1). None of the control group had medication history or relevant clinical signs and had no obvious abnormalities in the last 6 months.

**Table 1:** Descriptions of control and hypothyroid dogs (10 dogs per group).

No	Breed	Age (years)	Sex	BCS	Weight (kg)	Clinical signs
Control 1	Golden Retriever	6	MI	7/9	42.6	None
Control 2	Poodle	7	FS	5/9	5.3	None
Control 3	Siberian Husky	8	MI	6/9	35.2	None
Control 4	Welsh Corgi	4	FS	6/9	15.6	None
Control 5	Poodle	9	MC	6/9	6.7	None
Control 6	Poodle	13	MC	5/9	6.2	None
Control 7	Labrador Retriever	7	MC	7/9	40.2	None
Control 8	Mix	6	MI	6/9	9.7	None
Control 9	Schnauzer	7	MI	6/9	7.3	None
Control 10	Pekingese	8	MC	5/9	6.6	None
Treatment 1	Poodle	7	MI	7/9	7.1	Note^1^
Treatment 2	Golden Retriever	10	FS	6/9	32.0	Note^1^
Treatment 3	Poodle	13	MI	6/9	5.8	Note^1^
Treatment 4	Poodle	13	FS	5/9	4.3	Note^1^
Treatment 5	Mix	12	MC	8/9	20.4	Note^1^
Treatment 6	Poodle	9	MC	7/9	6.8	Note^1^
Treatment 7	Shiba Ken	4	MC	7/9	7.5	Note^1^
Treatment 8	Poodle	8	MI	6/9	5.8	Note^1^
Treatment 9	Border Collie	4	MI	7/9	28.5	Note^1^
Treatment 10	Poodle	5	MC	5/9	4.2	Note^1^

FS: Female dogs spayed; MI: Male dogs intact; MC: Male dogs castrated. Note^1^: see Table 2.

On day 0 (Table 2), all dogs with hypothyroidism appeared lethargic. Among them, seven dogs with hypothyroidism had a decreased basal metabolic rate, along with mental dullness and exercise intolerance. Three dogs had decreased appetite, two had decreased weight gain, and five were designated obese. Six dogs with hypothyroidism had skin lesions, including bilateral symmetric alopecia, poor coat quality, and hyperpigmentation. On day 14 (Table 2), five dogs with decreased basal metabolic rate had returned to normal activity and appetite and the remaining two were close to normal levels on day 45 (Table 2). Dogs with skin lesions had improvements, including slower shedding and growth of downy hair on the shedding area, with no further shedding and lighter coloration of the dermal hyperpigmentation, although skin lesions were still not completely healed by day 45.

**Table 2:** Typical clinical signs of hypothyroid (treatment group) dogs (*n* = 10).

Signs	Day 0	Day 14	Day 45
Decreased basal metabolic rate (*n* = 7)	Lethargy, weakness, mental dullness (*n* = 5) Exercise intolerance (*n* = 7) Decreased appetite (*n* = 3) Obesity (*n* = 5)	Returned to pre-disease levels in terms of activity and appetite (*n* = 5) Improved activity and appetite (*n* = 2)	Returned to pre-disease levels in terms of activity and appetite (*n* = 5) Close to pre-disease levels in terms of activity and appetite (*n* = 2)
Skin lesions (*n* = 6)	Alopecia with bilaterally symmetric. Truncal non-pruritic alopecia (*n* = 5) Poor coat quality (*n* = 2) Hyperpigmentation (*n* = 2)	Slower shedding and growth of downy hair (*n* = 5) Poor coat quality (*n* = 2) Hyperpigmentation (*n* = 2)	No further shedding (*n* = 5) Improved coat quality (*n* = 2) Lighter coloration of hyperpigmentated skin (*n* = 2)

### 3.2 CBC and serum biochemistry

The CBC and serum biochemistry data are provided in Supplementary Tables 1, 2. In the control group, neither CBC nor serum biochemistry had any abnormalities. In the treatment group, the CBC was normal but there were significant changes in biochemical cholesterol and triglycerides compared to the control group. On day 0, the treatment group dogs with hypothyroidism had hypercholesterolemia (mean serum cholesterol of 434.92 mg/dL) that was higher than the control group (*p* < 0.05) and the reference range. Furthermore, treatment group dogs with hypothyroidism had hypertriglyceridemia (mean serum triglycerides of 439.3 mg/dL), higher than the control group (*p* < 0.01) and the reference range. On day 14, in the treatment group, mean serum cholesterol and triglycerides had decreased to 318.41 mg/dL (within the reference range) and 346.3 mg/dL (above the reference range), respectively; although both serum cholesterol with hypercholesterolemia and triglycerides concentrations with hypertriglyceridemia had decreased (*p* < 0.05 and *p* < 0.01, respectively), but they remained higher than in the control group (*p* < 0.05 and *p* < 0.01). On day 45, the mean serum cholesterol had decreased to 214.43 mg/dL (*p* < 0.01); serum cholesterol in all the dogs with hypothyroidism was within the reference range; mean triglycerides had decreased to 247.0 mg/dL (above the reference range); and serum triglycerides concentrations with hypertriglyceridemia had decreased (*p* < 0.01) but remained higher than the control group (*p* < 0.01) at the end of the study.

### 3.3 T4, fT4, and TSH concentrations

**Figure 1:**
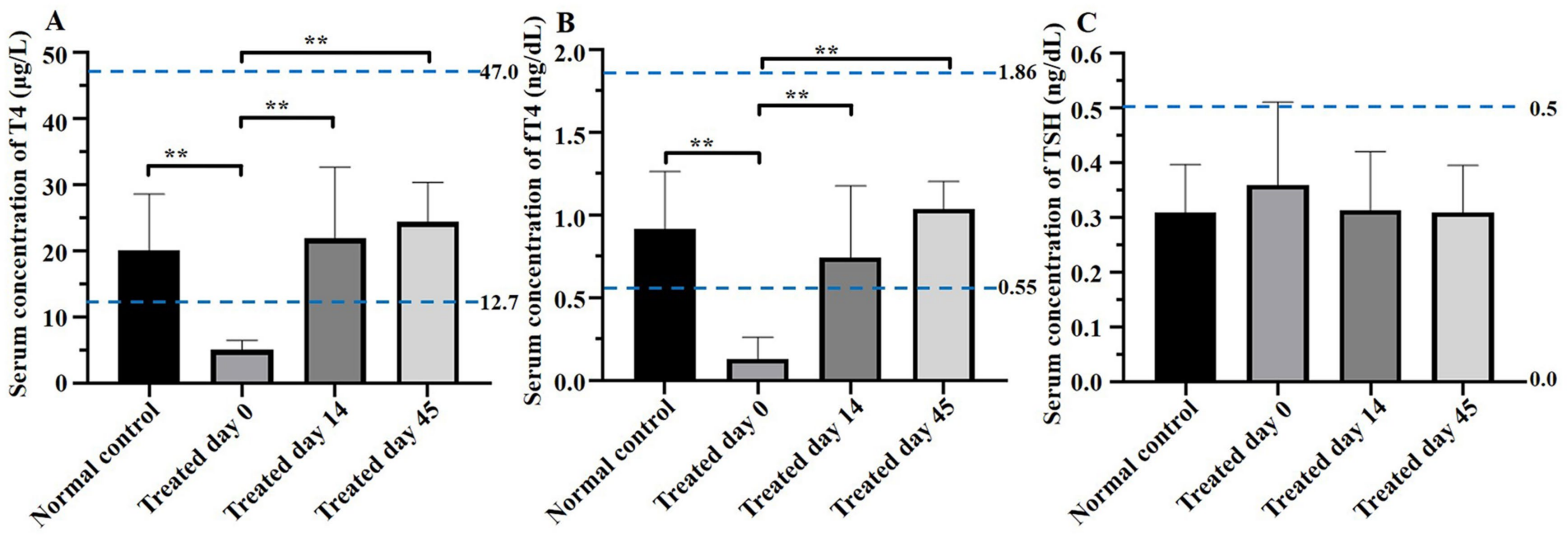
Comparisons of thyroxine (T4), free thyroxine (fT4), and thyroid-stimulating hormone (TSH) concentrations between groups on days 0, 14, and 45. On day 0, T4, and fT4 concentrations were lower (*p* < 0.01) and TSH concentration was higher in treatment versus control groups. In contrast, on days 14 and 45, both T4 and fT4 in the treatment group were significantly higher than on day 0 and not significantly different from the control and TSH concentrations in the treatment group were within the normal range. Blue dotted lines indicate the reference range. Asterisks indicate differences between groups (**p < 0.01).

Serum T4, fT4, and TSH concentrations given are in Table 3 and Figure 1. On day 0, T4 and fT4 concentrations were lower (*p* < 0.01) in treatment versus control groups. In contrast, on days 14 and 45, both T4 and fT4 in the treatment group were significantly higher than on day 0 but not significantly different from control. On day 0, TSH concentration was higher in the treatment group than in the control group, and two dogs with hypothyroidism had TSH concentrations above the reference range. However, on day 45, TSH concentrations in all dogs with hypothyroidism were within the normal range.

**Table 3:** Mean ± standard deviation for serum T4, fT4 and TSH concentrations in control dogs and those with hypothyroidism (10 dogs per group) for all time points during the experimentation period.

End point	Control group	Treatment group			Reference range
		Day 0	Day 14	Day 45	
T4 (μg/L)	20.14 ± 8.02	5.07 ± 1.31^**^	21.89 ± 10.20^**##**^	24.34 ± 5.58^**##**^	12.7–47.0
fT4 (ng/dL)	0.917 ± 0.329	0.144 ± 0.128^**^	0.895 ± 0.347^**##**^	1.039 ± 0.152^**##**^	0.55–1.86
TSH (ng/mL)	0.31 ± 0.08	0.36 ± 0.14	0.31 ± 0.10	0.31 ± 0.08	0–0.5

^**^p < 0.01; compared to treatment Day 0, ^##^*p* < 0.01.

### 3.4 SOD, CAT, and GSH-px activities and MDA concentrations

**Figure 2:**
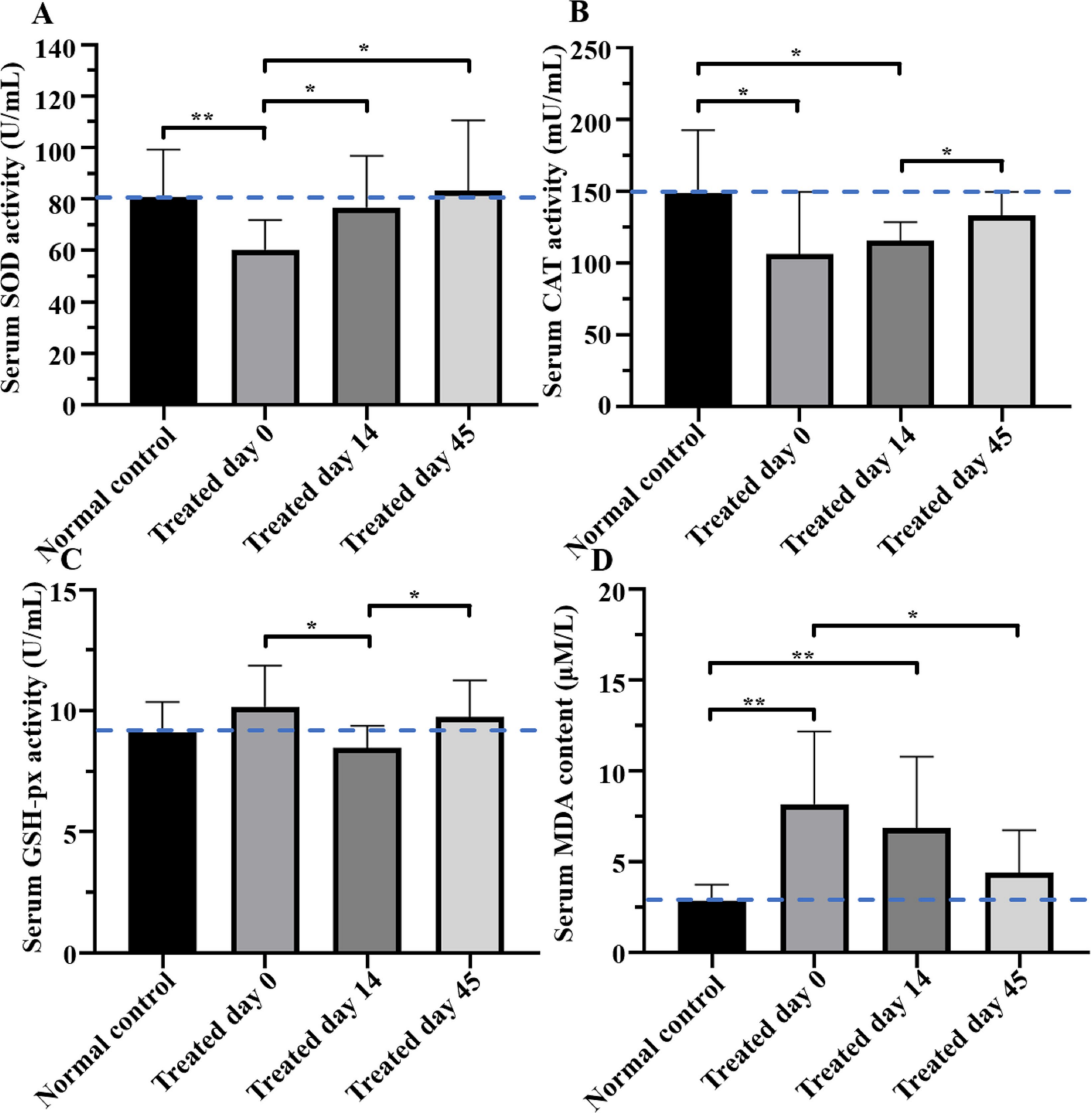
Comparisons of superoxide dismutase (SOD), catalase (CAT), and glutathione peroxidase (GSH-px) and malondialdehyde (MDA) concentrations between groups on day 0, 14, and 45. The SOD activity in the treatment group was lower than that in control group (*p* < 0.01), but it had increased on day 14 (*p* < 0.05) and day 45 (*p* < 0.05), and on the latter day was not different from the control group (*p* > 0.05). On both day 0 (*p* < 0.05) and day 14 (*p* < 0.05), CAT activity in the treatment group was significantly lower than that in the control group, and it had increased (*p* < 0.05) on day 45, but was still lower than that in the control group. GSH-px in the treatment group on day 14 was lower than that in the control group (*p* < 0.05). MDA in the treatment group was higher (*p* < 0.01) than that in the control group on days 0 and 14, whereas on day 45, MDA was decreased (*p* < 0.05) but remained higher than that in the control group. Blue dotted lines indicate the average concentration in the control group. Asterisks indicate differences between groups (*p < 0.05, **p < 0.01).

Serum activities of SOD, CAT, and GSH-px and MDA concentrations are provided in Table 4 and Figure 2. The SOD activity in the treatment group on day 0 was lower (*p* < 0.01) than that in the control group; however, it had increased on day 14 (*p* < 0.05) and day 45 (*p* < 0.05), and on the latter day, it was not significantly different from the control group (*p* > 0.05). On day 0 (*p* < 0.05) and day 14 (*p* < 0.05), the CAT activity in the treatment group was lower than that in the control group, but it had increased (*p* < 0.05) on day 45, although it was still lower than that in the control group. GSH-px in the treatment group on day 14 was lower than that in the control group. MDA levels in the treatment group were higher (*p* < 0.01) than that in the control group on days 0 and 14, whereas on day 45, MDA levels decreased (*p* < 0.05) but remained higher than that in the control group.

**Table 4:** Mean ± standard deviation for SOD, CAT, GSH-Px activities and MDA concentrations in control and hypothyroid dogs (10 dogs per group) for all time points during the experimentation period.

End point	Control group	Treatment group		
		Day 0	Day 14	Day 45
SOD (U/mL)	80.71 ± 17.52	60.22 ± 11.01^**^	76.69 ± 19.04^**#**^	83.27 ± 25.20^**#**^
CAT (mU/mL)	148.50 ± 41.56	105.88 ± 41.27^*^	115.45 ± 12.39^*^	132.89 ± 15.44^**#**^
GSH-P (U/mL)	9.11 ± 1.19	10.21 ± 1.55	8.41 ± 0.92^**#**^	9.73 ± 1.41
MDA (μmoL)	2.82 ± 0.86	8.16 ± 3.78^**^	6.82 ± 3.75^**^	4.38 ± 2.17^**#**^

*p < 0.05, **p < 0.01; compared to treatment Day 0, ^#^*p* < 0.05.

### 3.5 Association between thyroid and oxidative stress profiles

The association between thyroid and oxidative stress profiles in dogs with hypothyroidism on day 0 are provided in Table 5. In the treatment group, there were positive correlations between T4 and MDA (*r* = −0.5629) (*p* < 0.01) and between fT4 and MDA (*r* = −0.5964) (*p* < 0.01). There was also a negative correlation between T4 and CAT (*r* = 0.5899) (*p* < 0.01).

**Table 5:** Association of thyroid and oxidative stress profiles on day 0 in dogs with hypothyroidism.

Variables	SOD↓	CAT↓	GSH-Px↑	MDA↑
T4**↓**	*p* > 0.05	*p* = 0.0062	*p* > 0.05	*p* = 0.0098
	*r* = 0.2866 NS	*r* = 0.5899**	*r* = −0.3383 NS	*r* = −0.5629**
fT4**↓**	*p* > 0.05	*p* > 0.05	*p* > 0.05	*p* = 0.0055
	*r* = 0.4052 NS	*r* = 0.3489 NS	*r* = −0.3761 NS	*r* = −0.5964**
TSH**↑**	*p* > 0.05	*p* > 0.05	*p* > 0.05	*p* > 0.05
	*r* = −0.1400 NS	*r* = −0.1659 NS	*r* = 0.0052 NS	*r* = 0.2695 NS

NS = not significant (assessed by Spearman’s correlation analysis and correlation coefficient is represented as *r* value); ↓ = data decreased; ↑ = data increased. ** *p* < 0.01.

## 4 Discussion

### 4.1 Comparison of redox status on day 0

According to reports from the Chinese Veterinary Medical Association, there were approximately 51.75 million dogs in China at the end of 2023. The prevalence of canine hypothyroidism in China remains unclear, and the antioxidant status of dogs with hypothyroidism and its changes after levothyroxine replacement therapy are also poorly defined. Our objective was to confirm whether levothyroxine sodium could alleviate the hypothyroidism-induced damage by improving the disturbed redox balance. Herein, on day 0, SOD and CAT activities were lower, and GSH-px activity and MDA concentrations were higher in the treatment group compared to the control group. Generally, the enzymes SOD, CAT, and GSH-px, which are widespread animal peroxidases, and MDA, a major lipid peroxide, can be used as indicators to assess redox status (15). It was reported that canine hypothyroidism is associated with oxidative stress, manifested as elevations in MDA and TAC, and accompanied by hyperlipidemia (16). Furthermore, hypothyroid dogs experienced a significant decrease in SOD, catalase, and reduced glutathione levels compared to the healthy control group dogs (17). Increased ROS concentrations in hypothyroidism may result in a pro-oxidation environment, which could result in decreased antioxidant paraoxonase activity, with increased MDA and NO concentrations (18). As a result, lipid peroxidation may have a role in the pathogenesis of the atherosclerosis, as the oxidative marker values in hypothyroidism positive dogs were significantly increased as compared to the healthy control group. Indeed, hypothyroidism decreases metabolism and reduced synthesis and activities of SOD and CAT (10). This results in decreased antioxidant system function and a lower availability of ROS scavengers, which in turn elevates ROS levels and further inhibits SOD and CAT activities. Elevated ROS levels and accumulations of hydrogen peroxide and lipid peroxides cause a compensatory increase in GSH-px to scavenge the accumulated hydrogen peroxide and lipid peroxides (19). Elevated levels of GSH-px may also be associated with elevated levels of TSH (20). Elevated levels of MDA is associated with ROS accumulation and hypercholesterolemia and hyperlipidemia in dogs with hypothyroidism (21–24). The current results confirmed decreased antioxidant capacity in dogs with hypothyroidism.

The current findings were consistent with the perspective that hypothyroid patients have deficient antioxidant defense, with decreases in SOD and GSH activity, in addition to increases in GSH-px activity and MDA concentrations, indicating a reduced antioxidant status and oxidative stress in hypothyroid patients (10, 25). Canine hypothyroidism have alterations in serum redox status, with a significantly higher trolox equivalent antioxidant capacity, paraoxonase type 1, GSH-px, total oxidant status, SOD, and reactive oxygen-derived compounds, and a significantly lower advanced oxidation protein products in serum of dogs with hypothyroidism. Meanwhile, significantly lower ferric reducing ability of saliva and advanced oxidation protein products were observed in saliva of dogs with hypothyroidism. The only analyte showing significant changes was MDA which was significantly higher in dogs with hypothyroidism (26). On the one hand, data on hypothyroidism and oxidative stress in humans are conflicting. Serum SOD in hypothyroid patients was not significantly different from that in the healthy control group (18). On the other hand, overt hypothyroidism is associated with increased oxidative stress (11), as plasma SOD and CAT were significantly elevated in hypothyroid patients compared to healthy controls and were significantly associated with elevated levels of TSH. Conversely, SOD, CAT, and GSH-px were all significantly reduced in hypothyroid persons (12), and there was a reduction in antioxidative defense in patients with hypothyroidism.

### 4.2 Redox status post-levothyroxine sodium treatment

During the treatment period, SOD and CAT increased and MDA decreased, whereas GSH-px decreased and then increased in the treatment group. In the treatment group, T4 and fT4 on days 14 and 45 reached normal concentrations, which increased antioxidant status and decreased ROS production. The enhanced ability of the antioxidant system to scavenge ROS decreased MDA production, implying enhancement of antioxidant properties by levothyroxine sodium exceeded the inhibitory effect caused by increased ROS. MDA was positively correlated with low-density lipoprotein, cholesterol, total cholesterol, and triglycerides (21, 22); therefore, the reduction in MDA in the treatment group in this study may also have been related to improvements in hypercholesterolemia and hyperlipidemia. The decrease in GSH-px on day 14 in the treatment group, which was lower than that on day 0, may be related to the inhibitory effects of accumulated MDA, ROS, and hydrogen peroxide, as well as duration and severity of hypothyroidism. The effect of levothyroxine sodium on GSH-px at day 14 can be determined by this test alone. In contrast, GSH-px was elevated in the treatment group on day 45, indicating that the elevating effect of levothyroxine sodium on GSH-px was greater than the inhibitory effect of increased MDA and related ROS and hydrogen peroxide, and thus treatment significantly elevated GSH-px on day 45. Although the antioxidant properties of the treatment group were significantly higher on day 45, they were still lower than those of the control group. We inferred that levothyroxine did not completely mimic the physiological release of thyroid hormones, and that dogs with hypothyroidism remained in a state of oxidative stress (27).

Furthermore, current results indicated that levothyroxine replacement improved oxidative status in patients with primary hypothyroidism, based on significantly decreased concentrations of MDA and increased CAT activity (13). Increased MDA concentrations in patients represent increased lipid peroxidation, which may have an important role in the pathogenesis of the hypothyroidism. Increased oxidative stress in patients with hypothyroidism was overcome by levothyroxine treatment. MDA can be used as a reliable marker of oxidative stress and oxidative damage, along with other biomarkers of oxidative stress (28). However, in human primary hypothyroid patients, 75 μg/day levothyroxine sodium for 15 days caused a further elevation of plasma hydrogen peroxide levels, which exacerbated oxidative damage. Based on these collective findings, we inferred that levothyroxine sodium has a high effective treatment and can restore hypothyroidism-triggered alterations related to antioxidant capacity. Thus, it is a promising agent for the treatment of dogs with hypothyroidism.

## 5 Conclusion

In dogs with hypothyroidism, antioxidant properties were decreased with elevated oxidative stress. However, in dogs with hypothyroidism, levothyroxine sodium (0.02 mg/kg twice daily) for 45 days improved hypothyroidism and redox status of biomarkers in serum to some extent on day 14, with significant improvement on day 45.

## Data Availability

The original contributions presented in the study are included in the article/Supplementary material, further inquiries can be directed to the corresponding authors.
