# Supplementary material for: Redox status of biomarkers in serum of dogs with hypothyroidism and its treatment with levothyroxine sodium
Source: Front Vet Sci. 2025 Apr 4;12:1490369. doi: 10.3389/fvets.2025.1490369 (PMC12006059; doi:10.3389/fvets.2025.1490369)
Supplement: Supplementary file 1 [file Table_1.docx]

**SUPPLEMENTARY TABLE 1.** Mean ± standard deviation for complete blood count data in control and hypothyroid dogs (10 dogs per group)

| End point | Control group | Treatment group | | | Reference range |
| --- | --- | --- | --- | --- | --- |
|  |  | Day 0 | Day 14 | Day 45 |  |
| RBC (10^12/L) | 6.77±0.58 | 6.48±0.67 | 6.67±0.70 | 6.46±0.66 | 5.1-7.6 |
| HCT (%) | 45.9±4.0 | 43.9±5.0 | 44.3±6.1 | 43.6±3.6 | 35-52 |
| HGB (g/L) | 162±13 | 145±20 | 154±27 | 157±29 | 124-192 |
| MCV (fL) | 67.8±3.6 | 67.9±3.6 | 66.1±4.3 | 68.8±3.5 | 60-71 |
| MCH (pg) | 24.0±1.4 | 22.4±1.6 | 23.2±2.2 | 25.0±2.4 | 22-26 |
| MCHC (g/L) | 354±8 | 331±23 | 347±19 | 360±37 | 320-380 |
| RDW-CV (%) | 14.6±1.5 | 15.5±1.2 | 14.3±1.0 | 15.3±1.8 | 13.2-19.1 |
| RET-He (pg) | 23.3±1.0 | 21.2±1.9 | 21.9±1.6 | 22.3±1.2 | 22.3-29.6 |
| RET (%) | 0.71±0.40 | 1.05±0.50 | 0.87±0.29 | 0.69±0.30 | 0.3-2.4 |
| RET (K/uL) | 48.0±26.0 | 68.4±33.9 | 56.1±16.8 | 43.3±18.5 | 19.4-110 |
| NRBC (%) | 0.1±0.1 | 0.8±1.9 | 0.3±0.3 | 0.2±0.1 | 0-5 |
| NRBC (10^9/L) | 0.01±0.01 | 0.08±0.18 | 0.03±0.04 | 0.02±0.02 | - |
| WBC (10^9/L) | 9.37±1.48 | 9.92±2.21 | 11.06±2.79 | 9.93±1.87 | 5.6-18.4 |
| NEUT (%) | 73.5±11.7 | 65.1±9.2 | 74.1±11.6 | 62.2±10.4 | - |
| LYMPH (%) | 19.3±10.0 | 23.5±8.1 | 14.9±10.2 | 23.5±14.2 | - |
| MONO (%) | 5.0±1.3 | 7.7±2.6 | 8.8±4.0 | 13.0±8.1 | - |
| EO (%) | 2.1±1.4 | 3.6±2.7 | 2.1±1.3 | 3.5±1.1 | - |
| BASO (%) | 0.1±0.0 | 0.1±0.1 | 0.2±0.1 | 0.1±0.1 | - |
| NEUT (10^9/L) | 6.96±1.92 | 6.37±1.24 | 8.24±2.50 | 6.06±2.18 | 2.9-13.6 |
| LYMPH (10^9/L) | 1.75±0.86 | 2.37±1.13 | 1.53±1.01 | 2.12±1.22 | 1.1-5.3 |
| MONO (10^9/L) | 0.46±0.09 | 0.77±0.35 | 1.04±0.69 | 1.12±0.66 | 0.4-1.6 |
| EO (10^9/L) | 0.19±0.12 | 0.41±0.40 | 0.23±0.15 | 0.35±0.13 | 0.1-3.1 |
| BASO (10^9/L) | 0.01±0.00 | 0.01±0.01 | 0.02±0.02 | 0.01±0.01 | 0-0.1 |
| PLT (K/uL) | 335±85 | 294±53 | 399±125 | 379±86 | 148-484 |
| MPV (fL) | 10.0±1.0 | 11.0±1.6 | 9.9±1.1 | 9.9±0.8 | 9.1-12.7 |
| PCT (%) | 0.37±0.07 | 0.34±0.06 | 0.44±0.14 | 0.41±0.10 | 0.14-0.46 |
