# Supplementary material for: Redox status of biomarkers in serum of dogs with hypothyroidism and its treatment with levothyroxine sodium
Source: Front Vet Sci. 2025 Apr 4;12:1490369. doi: 10.3389/fvets.2025.1490369 (PMC12006059; doi:10.3389/fvets.2025.1490369)
Supplement: Supplementary file 2 [file Table_2.docx]

**SUPPLEMENTARY TABLE 2.** Mean ± standard deviation for serum biochemistry data in control and hypothyroid dogs (10 dogs per group)

| End point | Control group | Treatment group | | | Reference range |
| --- | --- | --- | --- | --- | --- |
|  |  | Day 0 | Day 14 | Day 45 |  |
| TBIL (mg/dL) | 0.16±0.02 | 0.15±0.00 | 0.15±0.00 | 0.16±0.01 | 0-0.80 |
| TP (g/dL) | 6.46±0.36 | 6.85±0.61 | 7.16±0.51 | 6.70±0.45 | 5.5-7.2 |
| ALB (g/dL) | 3.85±0.23 | 3.74±0.25 | 4.03±0.48 | 3.85±0.20 | 3.2-4.1 |
| GLB (g/dL) | 2.61±0.30 | 3.11±0.53 | 3.13±0.63 | 2.84±0.37 | 1.9-3.7 |
| A/G | 1.50±0.23 | 1.24±0.25 | 1.35±0.33 | 1.38±0.20 | 0.9-1.9 |
| GLU (mg/dL) | 99.91±7.95 | 99.57±9.88 | 99.83±9.04 | 87.49±6.22 | 68-104 |
| ALT (U/L) | 59.4±38.9 | 64.7±27.4 | 44.6±28.2 | 32.4±8.1 | 17-95 |
| AST (U/L) | 30.7±12.0 | 27.6±10.3 | 22.5±8.0 | 27.4±10.7 | 10-56 |
| ALP (U/L) | 66.5±37.4 | 66.9±41.1 | 71.6±28.2 | 58.6±33.0 | 7-115 |
| GGT (U/L) | 5.59±1.53 | 5.38±2.29 | 3.84±1.60^**^ | 5.30±1.74 | 0-8 |
| TBA (umol/L) | 8.7±14.4 | 9.1±9.6 | 3.8±3.6^**^ | 10.6±6.0 | 0-25 |
| UREA (mg/dL) | 41.18±13.88 | 45.67±14.88 | 46.41±12.16 | 47.23±11.17 | 19-55.7 |
| CREA (mg/dL) | 0.88±0.29 | 0.79±0.21 | 0.91±0.21 | 0.98±0.27 | 0.6-1.4 |
| P (mg/dL) | 3.64±1.06 | 4.23±0.43 | 3.78±0.61 | 3.47±0.91 | 2.7-5.4 |
| Ca (mg/dL) | 10.49±0.46 | 10.91±0.55 | 10.82±0.64 | 10.49±1.18 | 9.4-12.0 |
| CHOL (mg/dL) | 216.00±57.23 | 434.92±333.08^*^ | 318.41±71.06^#^ | 214.43±57.96^##^ | 136-392 |
| TG (mg/dL) | 75.9±43.7 | 439.3±299.2^**^ | 346.3±352.9^##^ | 247.0±313.5^##^ | 23-102 |
| CK (U/L) | 103.0±37.5 | 133.6±54.9 | 94.0±50.4 | 100.8±31.9 | 0-314 |
| K (mmol/L) | 4.88±0.27 | 4.99±0.49 | 5.09±0.26 | 4.87±0.37 | 4.1-5.4 |
| Na (mmol/L) | 146.0±3.0 | 146.0±5.0 | 144.0±2.0 | 148.0±4.0 | 143-150 |
| CL (mmol/L) | 113.0±4.3 | 111.3±3.6 | 109.8±2.7 | 115.1±2.6 | 106-114 |

**P* < 0.05, ***P* < 0.01; compared to treatment Day 0, ##*P* < 0.01.
